# Melatonin Alleviates Chilling Injury Symptom Development in Mango Fruit by Maintaining Intracellular Energy and Cell Wall and Membrane Stability

**DOI:** 10.3389/fnut.2022.936932

**Published:** 2022-06-30

**Authors:** Renu Bhardwaj, Morteza Soleimani Aghdam, Marino Bañon Arnao, Jeffrey K. Brecht, Olaniyi Amos Fawole, Sunil Pareek

**Affiliations:** ^1^Department of Agriculture and Environmental Sciences, National Institute of Food Technology Entrepreneurship and Management, Sonipat, India; ^2^Department of Horticultural Science, Imam Khomeini International University, Qazvin, Iran; ^3^Department of Plant Biology (Plant Physiology), Faculty of Biology, University of Murcia, Murcia, Spain; ^4^Department of Horticultural Sciences, University of Florida, Gainesville, FL, United States; ^5^Postharvest Research Laboratory, Department of Botany and Plant Biotechnology, University of Johannesburg, Johannesburg, South Africa

**Keywords:** cell wall-degrading, cold storage, intracellular energy, *Mangifera indica*, membrane integrity

## Abstract

The efficacy of the signaling molecule melatonin for alleviating chilling injury (CI) in mango (*Mangifera indica* L.) fruit was studied to investigate the potential role of membrane integrity, energy charge, and ripening-related changes in the development of CI, and its management by melatonin. ‘Langra’ and ‘Gulab Jamun’ cultivar mango fruit was immersed in 100 μM of melatonin before storage for 28 days at 5°C with weekly transfers to shelf life at 25°C. CI symptom development was associated with compositional and enzymatic aspects of textural changes, cell membrane deterioration, and chemical energy status. Melatonin-treated ‘Langra’ fruit exhibited very low CI (5 vs. 21%) while ‘Gulab Jamun’ fruit exhibited higher CI (36 vs. 38%) during 28 days of storage at 5 ± 1°C. Higher chilling tolerance in melatonin-treated ‘Langra’ was associated with lower softening, ascribed to lower cell wall degrading *exo-* and *endo-*polygalacturonase, pectinesterase, and endo-1,4-β-D-glucanase. In addition, lower membrane deteriorating-phospholipase D and lipoxygenase activity in melatonin-treated ‘Langra’ corresponded to lower palmitic and stearic acids and higher oleic, linoleic, and linolenic acids accumulation, thus, higher unsaturated/saturated fatty acids ratio. Additionally, there was a higher intracellular energy supply with melatonin, represented by a higher adenylate energy charge (AEC) arising from higher ATP and ADP and lower AMP accumulation, related to higher H^+^-ATPase, Ca^2+^-ATPase, succinate dehydrogenase, and cytochrome c oxidase activities. This study for the first time provides evidence, suggesting that melatonin alleviation of CI is related to the preservation of membrane integrity, thereby protecting the intracellular energy supply, and preserving cell wall integrity *via* impeding cell wall degrading enzyme activities.

## Introduction

Mango (*Mangifera indica*) is economically one of the most important fruits, possessing high nutritive value, pleasant flavor, and attractive color and aroma (1). The nutritional composition of mango includes the presence of bioactive components (carotenoids, phenolic compounds, and ascorbic acid), fibers, minerals, vitamins, and organic acids (2). The unique qualities of mango fruit have resulted in its worldwide production in nearly 90 countries (3). Mango is a tropical fruit with high perishability due to high respiration and ethylene production rates, a climacteric ripening pattern, and a tropical and sub-tropical nature, which limits postharvest life to 3–4 days under ambient conditions.

The low-temperature storage is the most commonly used technique to extend the shelf life and to assure the nutritional aspect and sensory quality of mangoes. Most Indian mango cultivars cannot tolerate temperatures below 13°C due to the development of chilling injury (CI); the cultivars ‘Langra’ and ‘Dashehari’ are exceptions to some extent, tolerating exposure to 8–9 C. Symptoms of CI include abnormal (uneven) softening and color development, peel darkening and pitting, poor flavor due to lack of aroma, and lenticel prominence (4–7). Therefore, methods that can alleviate CI may raise global marketing by reducing postharvest degradation and increasing shelf life.

Among all biological processes, bio-membrane alteration is considered the initiation event at the molecular level in CI (8). Further, this cell membrane alteration is partially related to the homeostasis of cellular energy status. Insufficient energy supply to meet the demand of physiological metabolism in stressful conditions will lead to excessive reactive oxygen species (ROS). The ROS damages the cellular membrane, resulting in exacerbated permeability *via* membrane fatty acids peroxidation, resulting in a lower unsaturated to saturated fatty acids (unSFA/SFA) ratio (9). Besides this, fruit softening, which is a characteristic feature of fruit ripening, is abnormal in fruit exposed to chilling temperature due to impeded activity of cell wall metabolizing enzymes, such as polygalacturonase (PG), pectin esterase (PE), and *endo*-1,4-β-D-glucanase (EGase) (10–13). Therefore, regulation of CI susceptibility in fruit and vegetables is related to a series of these mechanisms. Any innovation or chemical treatment that can mitigate these major causes of CI has the potential to promote chilling tolerance in horticultural crops.

In plants, melatonin (*N-*acetyl-5-methoxytryptamine) not only serves as a receptor-dependent signaling molecule but also exhibits a powerful receptor-independent ROS-scavenging cascade (14–16). Recently, postharvest preservation and quality maintenance of fruits and vegetables by MT have gained attention due to strong evidence for their role in delaying senescence and promoting tolerance against fungal decay (17–19). Furthermore, many studies have provided evidence that MT application demonstrates a prominent efficiency in conferring chilling tolerance in pomegranate (20, 21), tomato (22–24), peach (10, 25–27), cucumber (28), litchi (29, 30), bell pepper (31), plum (32), mango (4–7), and banana (33) fruits by (1) promoting endogenous MT accumulation accompanied by (2) promotion of reduced nicotinamide adenine dinucleotide phosphate (NADPH) oxidase enzyme activity and signaling H_2_O_2_ accumulation, (3) enhancing ROS protection and scavenging systems activity, (4) preserving membrane unsaturation status by higher expression of fatty acid desaturase (*FADs*) genes, along with (5) lower expression of phospholipase D (*PLD*) and lipoxygenase (*LOX*) genes and enzyme activities, (6) promoting oxidative pentose phosphate, shikimic acid and phenylpropanoid pathways activity, (7) enhancing arginase pathway activity by activating the ZAT2/6/12-CBF1 signaling pathway for triggering endogenous nitric oxide, polyamines, proline, and γ-aminobutyric acid (GABA) accumulation, (8) enhancing intracellular energy adenosine triphosphate (ATP) and reducing power of NADPH supply, and (9) activating GABA shunt pathway activity.

However, to the best of our knowledge, there has been no study that evaluated the effectiveness of MT application concerning mango fruit chilling tolerance in terms of cell wall modification, membrane lipid metabolism, and energy metabolism. Therefore, the present study was conducted to investigate the effect of exogenous MT treatment in relation to cultivar dependent variability of chilling tolerance due to (i) cell wall modification enzymes, (ii) membrane lipid metabolism, and (iii) energy metabolism of mango fruit stored at the chilling temperature of 5 ± 1°C for up to 28 days.

## Materials and Methods

### Mango Fruit and MT Treatment

Fruit of the mango (*Mangifera indica* L.) cultivars ‘Langra’ and ‘Gulab Jamun’ were used in this study. The fruit was physiologically mature but pre-ripe, having 29 ± 1 N firmness at harvest time; however; the data presented here is after 3 days of shelf life. The fruit was hand-harvested from an orchard located in Sonepat, Haryana, India. The harvested fruit was transported to the laboratory and fruit with any injury or defects was sorted out from the main lot. The sorted fruit was disinfected with 1% (v/v) sodium hypochlorite for 2 min followed by a random division of 300 fruit from each cultivar into six groups, consisting of 50 fruits in each group. The first three groups were immersed in distilled water (control) in three different buckets, and the other three groups were immersed in 100 μM MT (treated) for 2 h at 25 ± 2°C under the low light conditions in three different buckets (7). After air drying for 2 h at 25°C, the fruits were placed into storage at 5 ± 1°C and RH 85–90%. Observations were taken after 3 days of simulated shelf life at ambient temperature (25°C, 90–95% RH) on every 7 days interval during 28 days of storage. Each observation was recorded in triplicates and each replicate consisted of three fruits. For analyses, the fruit epicarp and mesocarp tissues were frozen with liquid nitrogen on the day of observation followed by storage at −80°C. Besides this, a lot of 45 fruits from each treatment were used for recording CI symptom development at ambient conditions (25°C, 90–95% RH) after 3 days of shelf life (7).

### Chilling Injury Index and Firmness Evaluation

Visual assessment based on surface symptoms in the form of skin darkening, pitting, and lenticel prominence was used for providing the ranking to CI as described by Concellón et al. (34) with some modifications. In this study, rank 0 indicates none, rank 1 indicates 1–20%, rank 2 indicates 21–40%, rank 3 denotes 41–60%, rank 4 denotes 61–80%, and rank 5 denotes 81–100% severity in CI symptoms over mango fruit. The assessment was done with fruit in triplicates, which were kept at ambient conditions for 3 days after their removal from cold storage. The CII was calculated as the sum of the product of rank given and the number of fruits in that particular scale divided by the total number of fruits considered in that observation.


$$C\,I\,I=\frac{\begin{array}{c} \sum (Rankscore\times Numberoffruitsrecievingthe\\ rankofCI) \end{array}}{T\,o\,t\,a\,l\,n\,u\,m\,b\,e\,r\,o\,f\,f\,r\,u\,i\,t\,s}$$

The firmness of fruit mesocarp was recorded with a texture analyzer (TA.HD Plus, Stable Micro Systems, United Kingdom) with 1.5 mm s^–1^ pre-test speed, 0.5 mm s^–1^ test speed, and 10 mm s^–1^ post-test speed. The probe used was 2 mm in diameter and the set penetration depth for observation at the equatorial region was taken as 5 mm. The fruit firmness was measured in triplicates on two opposite sides of the equatorial plane of each fruit.

### Cell Wall Degrading Enzymes Extraction and Activity Assessment

The extraction and assessment of activities of *exo-*PG (EC 3.2.1.82), *endo-*PG (EC 3.2.1.15), pectin esterase (PE; EC 3.1.1.11), and EGase (EC 3.2.1.4) were done according to Khan and Singh (35) with some modifications. For extraction, 13 g of fruit peel and pulp tissues were homogenized (IKA T18 Digital Ultra-Turrax, Cole-Parmer, India) separately with 13 ml of chilled 0.2% (w/v) sodium bisulphite (NaHSO_3_) and 12% (w/v) polyethylene-glycol (PEG). The homogenate was centrifuged (3-18KS, Sigma, Germany) at 12,000 × *g* at 4°C for 30 min, and the pellets were each washed with 4 ml of 0.2% (w/v) of NaHSO_3_ solution. The pellets obtained after re-centrifuging (3-18KS, Sigma, Germany) at 12,000 × *g* for 40 min were stored at −80°C and were used to determine the peel and pulp activities of *exo-*PG, *endo-*PG, PE, and EGase. For *exo-*PG and *endo-*PG, a pellet immersed in 50 mM sodium acetate buffer (pH 5.0) containing 0.5 M sodium chloride was incubated for 1 h at 4°C on an incubator shaker. Incubation was followed by centrifugation (3-18KS, Sigma, Germany) at 12,000 × *g* at 4°C for 15 min and the supernatant was diluted with 50-mM sodium acetate buffer (pH 5.0). The diluted supernatant was used as a crude extract for the determination of the activities of *exo-*PG and *endo-*PG.

For the *exo-*PG and *endo-*PG activities, mixtures consisting of 0.15 ml of 50 ml sodium acetate buffer (pH 4.4) containing 0.5% (w/v) of polygalacturonic acid (PGA) and 0.15 ml of an enzyme extract were incubated for 18 h at 30°C. An assay tube consisting of the above mixture, 0.1 M borate buffer (pH 9.0), and 1% (w/v) cyanoacetamide was boiled for 10 min, cooled, and the absorbance of the reaction mixture was measured at 274 nm with a spectrophotometer (Specord 200plus, Analytik Jena, Germany). The activity of the *exo-*PG enzyme was expressed in terms of μg of galacturonic acid mg^–1^ protein h^–1^ according to the standard curve of _*D*_-galacturonic acid. For *endo-*PG, 3 ml of an enzyme extract and chilled 2% (w/v) PGA in a 50-mM sodium acetate buffer (pH 4.4) were measured for viscosity change during 18 h of incubation period at 30°C with a viscometer (LABMAN Digital Rotational Viscometer, LMDV-60, India). The activity of *endo-*PG was expressed as a change in viscosity, i.e., Δ viscosity (mg^–1^ protein h^–1^) in triplicates with three fruits in each replication.

For PE activity, the pellet obtained through the above-mentioned extraction process was re-suspended in 15 ml of chilled 7.5% (w/v) sodium chloride, 0.75% (w/v) ethylenediaminetetraacetic acid (EDTA) solution at pH 6.5. After re-suspension, the mixture was incubated for 10 min at 4°C followed by centrifugation (3-18KS, Sigma, Germany) at 12,000 × *g* for 15 min. A total of 20 m of a citrus pectin solution (1%, w/v; pH 7.5) was mixed with 5 ml of the supernatant obtained as above, followed by titration with 0.01 N sodium hydroxide (NaOH). The activity of PE was estimated in triplicates (with three fruits in each replication) and was calculated from the total volume of NaOH solution required to maintain the pH of the reaction mixture up to 7.4 and expressed as mM NaOH mg^–1^ protein h^–1^.

The EGase activity was determined by stirring the extraction pellet in 15 ml of 0.1 M citrate-phosphate buffer (pH 6.0) consisting of 1 M NaCl followed by incubation for 1 h. The mixture was centrifuged (3-18KS, Sigma, Germany) at 12,000 × *g* for 15 min, and the supernatant obtained was mixed with 6 ml of 0.2% (w/v) carboxymethyl cellulose in citrate-phosphate buffer (pH 6.0). The activity of EGase was measured in triplicates with three fruits in each group. Estimation was done as a change in viscosity during 18 h of incubation at 30°C with a viscometer (LABMAN Digital Rotational Viscometer, LMDV-60, India) and was expressed as Δ viscosity (mg^–1^ protein h^–1^).

### Cell Membrane Deteriorating Enzymes Extraction and Activity Assessment

The activity of PLD (EC 3.1.1.4) was determined according to Liu et al. (36) involving some modifications. For extraction, 1 g of fruit tissue was homogenized (IKA T18 Digital Ultra-Turrax, Cole-Parmer, India) with 5 ml of chilled 100-mM Tris-HCl buffer (pH 7.0), followed by centrifugation (3-18KS, Sigma, Germany) at 12,000 × *g* at 4°C for 15 min. The supernatant obtained was used for PLD estimation. First, the reaction substrate was prepared by the addition of 0.4 g of lecithin (phosphatidylcholine) to 50 ml of ether followed by its evaporation using a rotary evaporator (Buchi, Germany) at 35°C. The dried solution was re-dissolved in 1 L of 100 mM of acetate buffer (pH 5.5) consisting of 5 mM of dithiothreitol (DTT) and 25 mM of calcium chloride. In an assay tube, 1 ml of enzyme extract was mixed with 3 ml of reaction substrate, followed by 1 h shaking at 28°C. The reaction substrate was washed with petroleum ether and the aqueous layer was collected. Furthermore, 2 g of Reinecke salt (NH_4_[Cr(NCS)_4_(NH_3_)_2_]⋅H_2_O) was dissolved in 100 ml of methanol and added to the reaction mixture in order to get a precipitate after centrifugation (3-18KS, Sigma, Germany) at 12,000 × *g* for 10 min. The precipitate was re-dissolved in 3 ml acetone, followed by spectrophotometric (Specord 200plus, Analytik Jena, Germany) analysis at 520 nm. The standard curve of choline chloride in 100-mM acetate buffer (pH 5.6) was used for estimation of PLD activity from triplicate samples (in which each replication consisted of three fruit), where 1 U was defined as mM of choline produced min^–1^.

For LOX (EC 1.13.11.12) enzyme, 1 g of fruit tissue was homogenized from triplicate samples (three fruit in each replication) with 5 ml of Tris-HCl buffer (100 mM, pH 7.0), followed by centrifugation (3-18KS, Sigma, Germany) at 10,000 × *g* at 4°C for 20 min. The LOX activity was assayed according to the method of Lin et al. (37) with some modifications. The standard assay mixture was prepared by mixing 200 μl Tween 20 and 40 μl linoleic acid in 40 m of sodium phosphate buffer (100 mM, pH 7.0). In a cuvette, 200 μl of LOX extract was added to a 1 ml standard assay mixture, and the absorbance was recorded (Specord 200plus, Analytik Jena, Germany) at 234 nm. The result was expressed in terms of U mg^–1^ protein, where U was defined as the amount of enzyme that causes an increase in absorption at 25°C by acting over linoleic acid.

### Fatty Acid Profiling (Unsaturated and Saturated)

The extraction of fatty acids was done in triplicates (three fruit in each replicate), according to Cao et al. (38), with some modifications. For the process of extraction, 20 g of fruit tissue was homogenized (IKA T18 Digital Ultra-Turrax, Cole-Parmer, India) with 10 ml of a mixture consisting of methanol, 0.1 M HCl, and water at a ratio of 200:100:1, followed by centrifugation (3-18KS, Sigma, Germany) at 12,000 × *g* for 10 min. The organic phase was dried, and methylation of fatty acids was done at boiling temperature with the addition of 1 ml of 140 M boron trifluoride dissolved in methanol. Furthermore, extraction of methylated fatty acids was done with hexane, again taken to dryness, followed by re-dissolving in 200 μl of chloroform prior to injection. Separation and quantification of fatty acids were done according to Mirdehghan et al. (39) with some modifications, using gas chromatography (Shimadzu GC-MS-TQ 8040, Kyoto, Japan).

### Estimation of Ca^2+^-ATPase and H^+^-ATPase Activities

The preparation of the extract for determination of Ca^2+^-ATPase (EC 7.2.2.10) and H^+^-ATPase (EC 3.6.3.6) activities was done in triplicates (each replication consisting of three fruit), according to Jin et al. (40), incorporating some modifications. The extraction buffer consisting of 50 mM Tris-HCl buffer (pH 8.0), 0.25 M sucrose, 0.3 M mannite, 0.5 g L^–1^ polyvinyl pyrrolidone, and 1 M EDTA was prepared. Afterward, 1 g of fruit tissue was homogenized (IKA T18 Digital Ultra-Turrax, Cole-Parmer, India) with 10 ml of pre-cooled extraction buffer. The homogenate was centrifuged (3-18KS, Sigma, Germany) at 5,000 × *g* at 4°C for 10 min, and the obtained supernatant was collected. The remaining sediment was washed with washing buffer prepared from 10-mM Tris-HCl, 0.25-M sucrose, 1-mM EDTA, and 0.3 M mannite. Finally, the sediment in dissolution with washing buffer was used as an enzyme extract.

For the assessment of Ca^2+^-ATPase and H^+^-ATPase, the reaction mixtures were prepared and the procedure followed was as per Jin et al. (40) with some modification. The reaction mixture of Ca^2+^-ATPase consists of Tris-HCl buffer (30 mM, pH 8.0), 3 mM magnesium sulfate (Mg_2_SO_4_), 0.1 mM sodium orthovanadate (Na_3_VO_4_), 50 mM sodium nitrate (NaNO_3_), 0.1 mM ammonium molybdate, 3 mM calcium nitrate [Ca(NO_3_)_2_], and an enzyme extract. On the other hand, the H^+^-ATPase reaction mixture consists of all the above mentioned constituents except 3 mM Ca(NO_3_)_2_. An assay tube consisting of a reaction mixture was added with 100 μl of 30-mM ATP-Tris (pH 8.0), followed by 20 min incubation at 37°C. Afterward, the reaction was terminated with the addition of 30 mM trichloroacetic acid (TCA), and absorbance was recorded at 660 nm with a spectrophotometer (Specord 200plus, Analytik Jena, Germany), and 1 unit of activity was expressed as a release of 1 μM phosphorus min^–1^.

### AMP, ADP, and ATP Measurement and Adenylate Energy Charge Calculation

The measurement of AMP, ADP, ATP, and AEC was done according to Yi et al. (41) with some modifications. For extract preparation, 2 g of fruit tissue was homogenized (IKA T18 Digital Ultra-Turrax, Cole-Parmer, India) with 5 ml of 0.6 M perchloric acid, followed by centrifugation (3-18KS, Sigma, Germany) at 12,000 × *g* at 4°C for 20 min. The supernatant volume was immediately adjusted to have a pH between 6.5–6.8 using potassium hydroxide solution. The final volume of supernatant was maintained at 4 ml and passed through a 0.45-μm filter (Millipore, HiMedia, India). Furthermore, AMP, ADP, and ATP were examined by high-performance liquid chromatography (HPLC) (Waters Corporation, United States) using a reserved-phase C18 column (Waters 80 Å, 5 μm particle size, 4.6 mm × 250 mm), accompanied by ultraviolet detector at wavelength 254 nm. Mobile phase A consisted of 0.04 M potassium dihydrogen phosphate and 0.06 M dipotassium hydrogen phosphate dissolved in distilled water and maintained at pH 7.0 using 0.1 M potassium hydroxide, whereas acetonitrile was used as mobile phase B. Programming consisted of a linear gradient program with 75–100% and 0–25% of mobile phase A and B, respectively, for 7 min at a flow rate 1.2 ml min^–1^. Lastly, the sample (10 μl) was injected into the HPLC for analysis of AMP, ADP, and ATP as per the external standard program. The energy charge was calculated from triplicates (consisting of three fruits in each replication) as ATP^+^1/2 ADP divided by the summation of AMP, ADP, and ATP.

### Protein Estimation

Protein estimation was done using the Bradford (42) method with some modifications. For extraction, 1 g of fruit tissue was homogenized (IKA T18 Digital Ultra-Turrax, Cole-Parmer, India) with 50 mM sodium phosphate buffer consisting of 0.5 mM magnesium chloride, 2 mM phenylmethylsulfonyl fluoride (PMSF), and polyvinylpyrrolidone (PVP). The homogenate was centrifuged (3-18KS, Sigma, Germany) at 12,000 × *g* at 4°C for 15 min and the supernatant obtained was used as the extract for protein determination. Furthermore, 0.1 ml of extract was mixed with 5 ml of Bradford reagent and was examined spectrophotometrically (Specord 200plus, Analytik Jena, Germany) at 595 nm after 5 min of incubation at ambient conditions. Bovine serum albumin (BSA) was used as the standard to estimate protein content in terms of mg g^–1^ fresh weight (FW).

### Statistical Analysis

Analysis of variance (ANOVA) was used for interpretation of the experimental results using a completely randomized design in triplicates. Additionally, the means were further compared using Duncan’s Multiple Range Test using SPSS (Version 20) with a 0.05 level of significance.

## Results

### Chilling Injury Index

**FIGURE 1:**
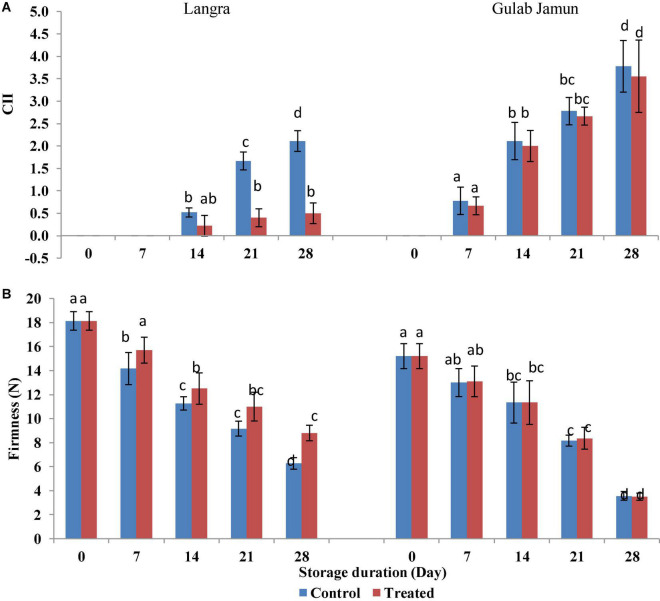
**(A)** Chilling injury index (CII), **(B)** firmness in ‘Langra’ and ‘Gulab Jamun’ mango fruit treated with 0 μM (control) or 100 μM (treated) MT for 2 h followed by storage at 5 ± 1°C. Readings were taken on every 7 days of 5 ± 1°C storage followed by 3 days of shelf life at room temperature (25°C, 90–95% RH). Each value is the mean for three replicates and vertical bars indicate the standard error. Error bars with different small letters on the same storage period show a significant difference (*P* ≤ 0.05).

The visual symptoms of CI, such as pitting, blackening, wrinkling, and browning over the peel of mango fruit, were regarded as CI visual signs, which were observed on the third day of observation at ambient conditions after removal from cold storage (5 ± 1°C) at every 7-day interval. ‘Langra’ mangoes without MT treatment exhibited CI symptoms just after 7 days of cold storage. However, MT-treated ‘Langra’ mangoes exhibited the first signs of CI on 14 days of storage (Figure 1A).

### Firmness

Decreasing trends in fruit firmness were seen with increasing storage duration. However, MT treatment exerted a positive effect in maintaining higher firmness of ‘Langra’ mangoes (Figure 1B). Whereas, no such effect was seen in ‘Gulab Jamun’ mangoes with exogenous MT application.

### Cell Wall Degrading Enzymes Activities

**FIGURE 2:**
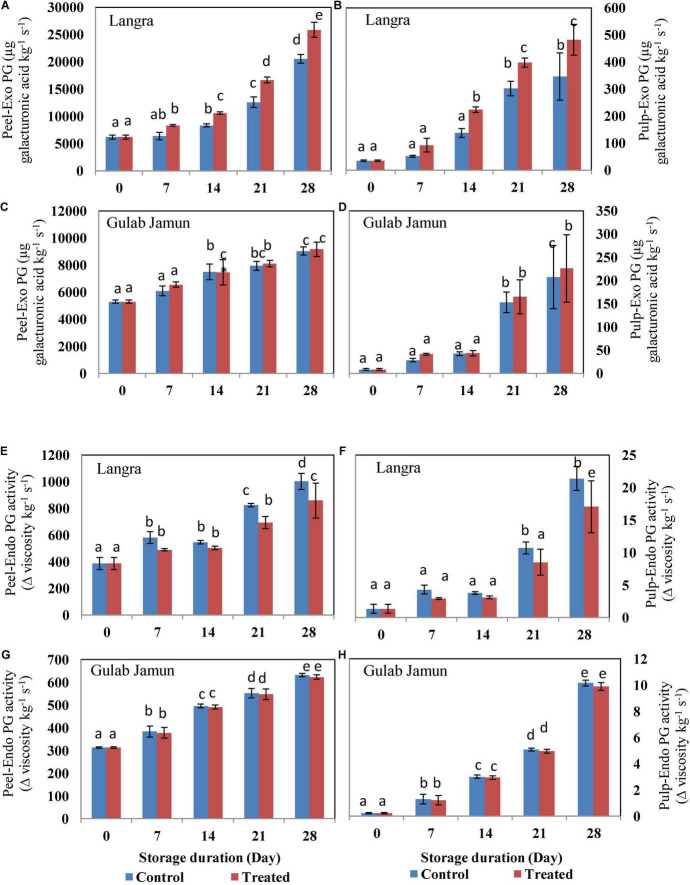
*Exo*-polygalacturonase (*Exo*-PG) activity in **(A)** ‘Langra’ peel, **(B)** ‘Langra’ pulp, **(C)** ‘Gulab Jamun’ peel, and **(D)** ‘Gulab Jamun’ pulp and *endo*-polygalacturonase (*Endo*-PG) activities in **(E)** ‘Langra’ peel, **(F)** ‘Langra’ pulp, **(G)** ‘Gulab Jamun’ peel, and **(H)** ‘Gulab Jamun’ pulp treated with 0 μM (control) or 100 μM (treated) MT for 2 h, followed by 28 days of storage at 5 ± 1°C and 3 days of shelf life at room temperature (25°C, 90–95% RH). Measurements were taken on every 7 days of storage. Each value is the mean of three replicates ± standard error. Error bars with different small letters on the same storage period show a significant difference (*P* ≤ 0.05).

**FIGURE 3:**
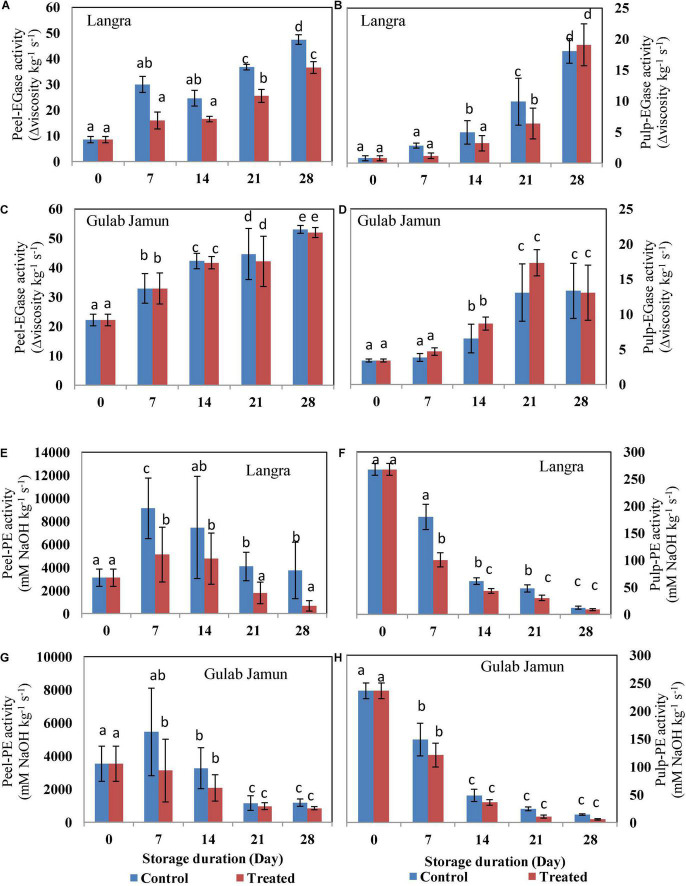
*Endo*-1,4-β-D-glucanase (EGase) activity in **(A)** ‘Langra’ peel, **(B)** ‘Langra’ pulp, **(C)** ‘Gulab Jamun’ peel, and **(D)** ‘Gulab Jamun’ pulp and pectin esterase (PE) activity in **(E)** ‘Langra’ peel, **(F)** ‘Langra’ pulp, **(G)** ‘Gulab Jamun’ peel, and **(H)** ‘Gulab Jamun’ pulp treated with 0 μM (control) or 100 μM (treated) MT for 2 h, followed by 28 days of storage at 5 ± 1°C and 3 days of shelf life at room temperature (25°C, 90–95% RH). Measurements were taken on every 7 days of storage. Each value is the mean of three replicates ± standard error. Error bars with different small letters on the same storage period show a significant difference (*P* ≤ 0.05).

The activities of fruit softening enzymes in the peel and pulp of the studied mango cultivars were positively affected by the progressive storage duration. The *exo-*PG (Figures 2A–D), *endo-*PG (Figures 2E,F), and EGase (Figures 3A–D) showed increasing trends as the duration of storage increased irrespective of the cultivar. Whereas, PE showed a somewhat decreasing trend with increasing storage duration. However, exogenous MT application was successful in suppressing the activity of *exo-*PG, *endo-*PG, and EGase, but in a cultivar dependent manner. Here, MT-treated peel and pulp of ‘Langra’ mangoes showed significant (*p* < 0.05) suppression in *exo-*PG (Figures 2A,B), *endo-*PG (Figures 2E,F), EGase (Figures 3A,B), and PE (Figures 3E,F) activities on one or another day. On the contrary, MT treatment resulted in increased activity of *exo-*PG (Figures 2C,D) and EGase (Figures 3C,D) in ‘Gulab Jamun’ mangoes; whereas no effect of MT application was noticed for *endo-*PG (Figures 2G,H) activity in ‘Gulab Jamun’ mangoes.

### Cell Membrane Deteriorating Enzymes Activity

**FIGURE 4:**
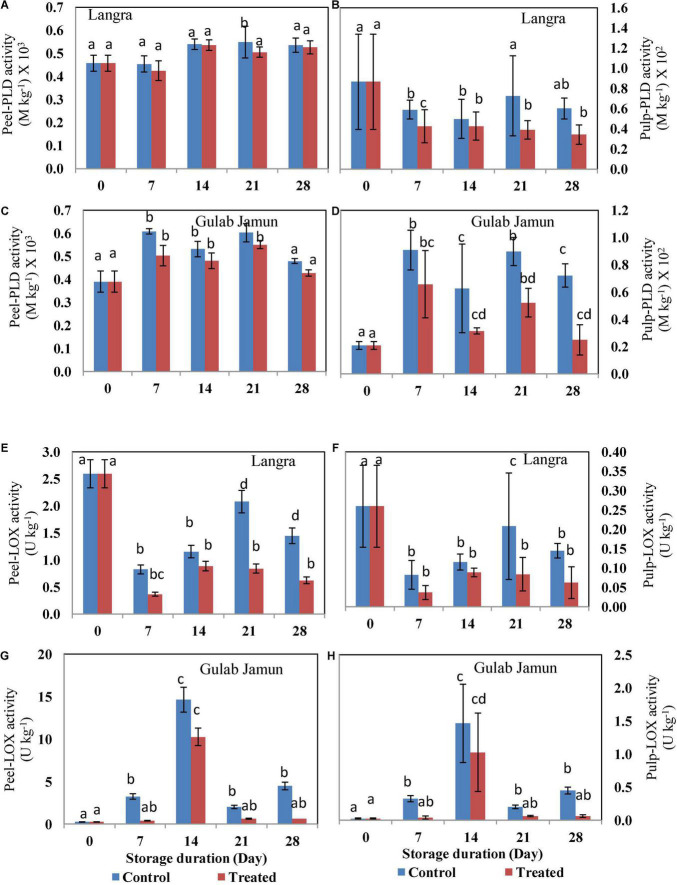
Phospholipase D (PLD) activity in **(A)** ‘Langra’ peel, **(B)** ‘Langra’ pulp, **(C)** ‘Gulab Jamun’ peel, and **(D)** ‘Gulab Jamun’ pulp and lipoxygenase (LOX) activity in **(E)** ‘Langra’ peel, **(F)** ‘Langra’ pulp, **(G)** ‘Gulab Jamun’ peel, and **(H)** ‘Gulab Jamun’ pulp treated with 0 μM (control) or 100 μM (treated) MT for 2 h, followed by 28 days of storage at 5 ± 1°C and 3 days of shelf life at room temperature (25°C, 90–95% RH). Measurements were taken on every 7 days of storage. Each value is the mean of three replicates ± standard error. Error bars with different small letters on the same storage period show a significant difference (*P* ≤ 0.05).

Phospholipase (PLD) is a primary enzyme involved in phospholipid degradation and is thereby involved in the integrity and functioning of biomembranes during environmental stress. However, no definite trend was observed in the activity of the PLD enzyme throughout the duration of 28 days at low-temperature storage (Figures 4A–D). The activity of PLD in control fruit was higher than in MT-treated mango peel and pulp for both the ‘Langra’ (Figures 4A,B) and ‘Gulab Jamun’ (Figures 4C,D) cultivars. Lower LOX activity was observed in the peel and the pulp of MT-treated ‘Langra’ (Figures 4E,F) and ‘Gulab Jamun’ (Figures 4G,H) mangoes.

### Membrane Unsaturation Status

Major amounts of five fatty acids were detected in the peel and pulp (Table 1) of mango fruit including two saturated fatty acids (palmitic acid and stearic acid) and three unsaturated fatty acids (palmitoleic acid, oleic acid, and linoleic acid). Palmitic acid and stearic acid showed rising trends (Table 1). However, fruit treated with MT had a lower accumulation of palmitic and stearic acids in both the peel and pulp of both cultivars with significant differences (*p* < 0.05) in ‘Langra’ mangoes. On the contrary, palmitoleic acid, oleic acid, and linoleic acid showed decreasing trends in ‘Langra’ mangoes in contrast to ‘Gulab Jamun’ mangoes. Additionally, ‘Langra’ mangoes treated with MT maintained significantly (*p* < 0.05) higher palmitoleic, oleic, and linoleic acid accumulation in their peel or pulp. Furthermore, the unsaturated to saturated fatty acid (unSFA/SFA) ratio was prominently higher in the peel and the pulp of MT-treated ‘Langra’ mangoes in contrast to ‘Gulab Jamun’ mangoes (Table 1). Therefore, treating mango fruit with MT may be advantageous in terms of unsaturated fatty acid maintenance during low temperature storage.

**TABLE 1:** Palmitic acid, stearic acid, palmitoleic acid, oleic acid, linoleic acid, and unsaturated/saturated fatty acid (USFA/SFA) in peel and pulp of ‘Langra’ and ‘Gulab Jamun’ mangoes treated with 0 μM (control) or 100 μM (treated) melatonin for 2 h followed by low temperature storage at 5 ± 1°C.

Parameter	Day	Langra				Gulab Jamun			
		Peel		Pulp		Peel		Pulp	
		0 μ M	100 μ M	0 μ M	100 μ M	0 μ M	100 μ M	0 μ M	100 μ M
Palmitic acid	0	2.82 ± 0.41 a	2.82 ± 0.41 a	22.35 ± 1.32 a	22.35 ± 1.32 a	3.84 ± 0.56 a	3.84 ± 0.56 a	19.11 ± 0.43 a	19.11 ± 0.43 a
	7	4.95 ± 1.89 a	3.19 ± 1.24 a	30.08 ± 0.58 b	23.00 ± 2.47 ab	6.54 ± 2.04 a	8.67 ± 0.93 ab	23.64 ± 0.70 a	21.96 ± 1.29 a
	14	9.90 ± 3.79 ab	6.38 ± 2.48 a	38.21 ± 0.80 c	60.10 ± 1.93 d	13.09 ± 4.08 ab	17.33 ± 1.86 bc	41.29 ± 3.36 b	27.01 ± 2.09 b
	21	19.08 ± 3.38 bc	18.09 ± 1.99 b	60.10 ± 1.93 d	45.39 ± 3.50 c	13.33 ± 3.92 ab	13.07 ± 3.93 abc	29.20 ± 0.87 b	28.61 ± 1.119 bc
	28	28.63 ± 5.08 c	27.13 ± 2.98 c	69.88 ± 2.22 e	64.03 ± 2.87 d	19.99 ± 5.89 b	19.60 ± 5.89 c	33.00 ± 1.91 c	32.57 ± 1.09 c
Stearic acid	0	1.86 ± 0.08 a	1.86 ± 0.08 a	0.93 ± 0.06 a	0.93 ± 0.06 a	0.36 ± 0.05 a	0.36 ± 0.05 a	0.51 ± 0.07 a	0.51 ± 0.07 a
	7	2.24 ± 0.10 a	1.38 ± 0.16 a	4.03 ± 0.18 b	2.48 ± 0.30 ab	0.43 ± 0.06 a	0.42 ± 0.04 a	0.78 ± 0.10 a	0.76 ± 0.07 a
	14	4.03 ± 0.18 b	2.48 ± 0.30 a	7.16 ± 0.30 c	5.44 ± 0.48 b	0.78 ± 0.10 a	0.76 ± 0.07 a	2.97 ± 0.66 a	2.75 ± 0.40 a
	21	7.16 ± 0.30 c	5.44 ± 0.48 b	8.68 ± 1.03 c	6.23 ± 1.57 b	2.97 ± 0.66 b	2.75 ± 0.40 b	4.07 ± 1.31 ab	3.73 ± 1.23 ab
	28	8.68 ± 1.03 d	6.23 ± 1.57 b	14.76 ± 1.75 d	10.60 ± 2.67 c	4.07 ± 1.31 b	3.73 ± 1.23 b	6.91 ± 2.22 b	6.35 ± 2.09 b
Palmitoleic acid	0	13.19 ± 151 a	13.19 ± 151 a	4.03 ± 0.52 a	4.03 ± 0.52 a	13.93 ± 0.93 a	13.93 ± 0.93 a	3.09 ± 0.24 a	3.09 ± 0.24 a
	7	8.10 ± 1.05 b	10.90 ± 1.66 a	1.11 ± 0.20 b	2.41 ± 0.61 b	6.69 ± 1.32 b	8.96 ± 1.32 b	0.88 ± 0.14 b	1.22 ± 0.14 b
	14	5.20 ± 0.40 bc	7.33 ± 0.84 b	0.74 ± 0.13 b	1.60 ± 0.41 bc	6.42 ± 0.56 bc	7.74 ± 0.52 b	0.58 ± 0.10 bc	0.82 ± 0.09 bc
	21	2.97 ± 1.06 cd	4.82 ± 0.20 bc	0.46 ± 0.08 b	1.00 ± 0.25 c	4.10 ± 0.26 cd	4.25 ± 0.25 c	0.37 ± 0.06 c	0.51 ± 0.06 c
	28	1.81 ± 0.23 d	3.24 ± 0.42 c	0.39 ± 0.07 b	0.84 ± 0.21 c	2.18 ± 0.38 d	2.68 ± 0.53 c	0.30 ± 0.05 c	0.42 ± 0.05 c
Oleic acid	0	37.01 ± 1.43 a	37.01 ± 1.43 a	9.77 ± 0.33 a	9.77 ± 0.33 a	16.28 ± 0.54 a	16.28 ± 0.54 a	8.78 ± 0.68 a	8.78 ± 0.68 a
	7	27.24 ± 2.10 a	36.06 ± 1.16 b	7.52 ± 0.25 b	9.55 ± 0.20 a	14.83 ± 2.55 a	15.19 ± 1.90 ab	6.75 ± 0.52 b	10.32 ± 0.84 b
	14	22.56 ± 0.76 b	28.66 ± 0.60 c	5.75 ± 0.62 c	7.52 ± 0.15 b	14.59 ± 4.52 a	14.97 ± 5.68 ab	5.49 ± 0.32 bc	5.91 ± 0.18 c
	21	15.04 ± 0.51 c	19.11 ± 0.40 d	5.00 ± 0.54 c	6.54 ± 0.13 c	13.50 ± 1.04 b	13.98 ± 3.37 bc	4.77 ± 0.28 c	5.14 ± 0.15 c
	28	11.50 ± 1.24 d	15.04 ± 0.29 d	3.45 ± 0.37 d	4.51 ± 0.09 d	10.98 ± 0.64 b	11.82 ± 0.35 c	3.29 ± 0.19 d	3.54 ± 0.11 d
Linoleic acid	0	5.44 ± 0.48 a	5.44 ± 0.48 a	1.85 ± 0.18 a	1.85 ± 0.18 a	2.75 ± 0.40 a	2.75 ± 0.40 a	1.65 ± 0.12 a	1.65 ± 0.12 a
	7	3.28 ± 0.29 b	4.08 ± 0.07 b	1.59 ± 0.07 ab	1.78 ± 0.13 a	1.66 ± 0.24 b	1.66 ± 0.24 b	1.27 ± 0.09 b	1.41 ± 0.09 ab
	14	2.48 ± 0.30 b	4.03 ± 0.18 bc	1.34 ± 0.06 bc	1.65 ± 0.13 a	0.76 ± 0.07 c	0.78 ± 0.10 c	1.22 ± 0.11 b	1.29 ± 0.11 ab
	21	2.06 ± 0.25 b	3.36 ± 0.15 c	1.10 ± 0.07 cd	1.52 ± 0.13 a	0.64 ± 0.06 c	0.65 ± 0.09 c	0.89 ± 0.08 c	1.06 ± 0.14 bc
	28	0.93 ± 0.06 c	1.67 ± 0.45 d	0.92 ± 0.04 d	1.55 ± 0.07 a	0.51 ± 0.07 c	0.74 ± 0.04 c	0.74 ± 0.08 c	0.79 ± 0.13 c
unSFA/SFA	0	11.96 ± 0.71 a	11.96 ± 0.71 a	0.67 ± 0.01 a	0.67 ± 0.01 a	8.10 ± 1.12 a	8.10 ± 1.12 a	0.69 ± 0.06 a	0.69 ± 0.06 a
	7	6.35 ± 2.11 b	13.23 ± 3.96 a	0.3 ± 0.01 b	0.55 ± 0.06 b	4.57 ± 2.29 ab	2.88 ± 0.18 b	0.36 ± 0.02 b	0.57 ± 0.02 b
	14	2.51 ± 0.72 c	5.24 ± 1.36 b	0.17 ± 0.01 c	0.30 ± 0.02 c	1.72 ± 0.29 bc	1.28 ± 0.17 bc	0.17 ± 0.01 c	0.27 ± 0.01 c
	21	0.80 ± 0.15 c	1.18 ± 0.10 b	0.10 ± 0.01 d	0.18 ± 0.01 d	1.32 ± 0.40 bc	1.26 ± 0.27 bc	0.18 ± 0.01 c	0.21 ± 0.02 cd
	28	0.39 ± 0.06 c	0.62 ± 0.08 b	0.06 ± 0.01 e	0.09 ± 0.01 d	0.64 ± 0.15 c	0.76 ± 0.20 c	0.11 ± 0.02 c	0.12 ± 0.01 d

*Measurements were taken on every 7 days of 5 ± 1°C storage followed by 3 days of shelf life at room temperature (25°C, 90–95% RH). Each value is the mean for three replicates. Values followed by different small letters in the same column show a significant difference (p ≤ 0.05) within the same storage period.*

### Energy Providing Enzyme Activities

**FIGURE 5:**
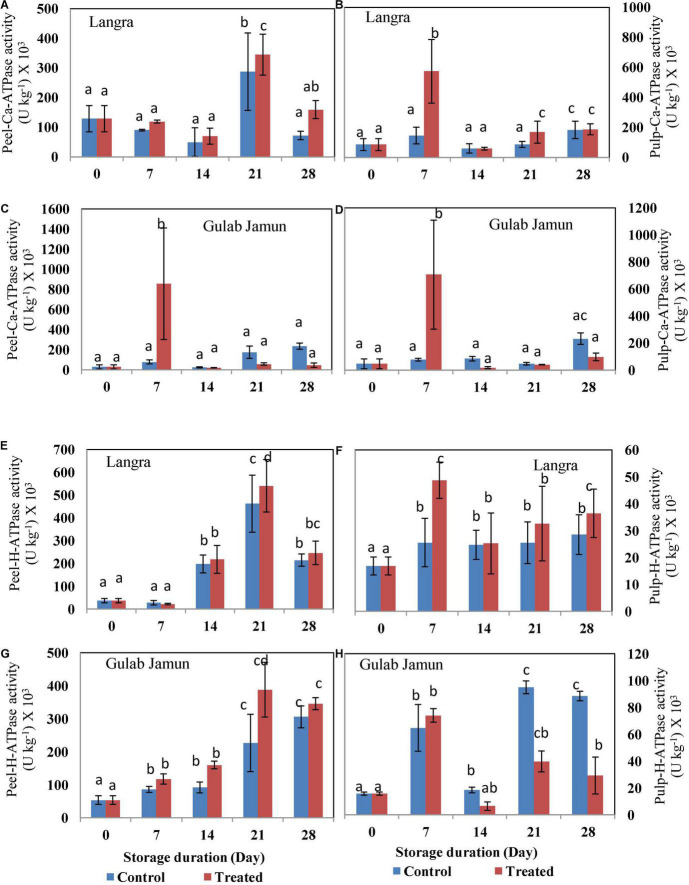
Ca^2+^-ATPase activity in **(A)** ‘Langra’ peel, **(B)** ‘Langra’ pulp, **(C)** ‘Gulab Jamun’ peel, and **(D)** ‘Gulab Jamun’ pulp and H^+^-ATPase activity in **(E)** ‘Langra’ peel, **(F)** ‘Langra’ pulp, **(G)** ‘Gulab Jamun’ peel and **(H)** ‘Gulab Jamun’ pulp treated with 0 μM (control) or 100 μM (treated) MT for 2 h, followed by 28 days of storage at 5 ± 1°C and 3 days of shelf life at room temperature (25°C, 90–95% RH). Measurements were taken on every 7 days of storage. Each value is the mean of three replicates ± standard error. Error bars with different small letters on the same storage period show a significant difference (*P* ≤ 0.05).

The activity of Ca^2+^-ATPase for both the cultivars followed an uneven pattern throughout the 28-day duration of storage (Figures 5A–D). Exogenous application of MT maintained higher activity of Ca^2+^-ATPase in both peel and pulp of ‘Langra’ mangoes (Figures 5A,B). However, peel (Figure 5C) and pulp (Figure 5D) of ‘Gulab Jamun’ mangoes maintained higher Ca^2+^-ATPase activity only on day 7 of observation, afterward MT-treated fruit were observed to have lower Ca^2+^-ATPase activity. Similarly, an apparent rise in the activity of H^+^-ATPase with MT treatment was observed in the peel (Figure 5E) and pulp (Figure 5F) of ‘Langra’ mangoes; whereas, some inconsistent rise and fall were observed in the peel (Figure 5G) and pulp (Figure 5H) of ‘Gulab Jamun’ mangoes.

### Intracellular Energy Status

The trend seen in ADP and ATP accumulation was one of decline over time while AMP accumulation increased as time progressed (Table 2). A significant difference (*p* < 0.05) was observed in the peel and pulp ADP and ATP accumulation of MT-treated ‘Langra’ mangoes only. However, a relatively higher AMP and AEC accumulation were noted with MT application in ‘Langra’ mangoes (Table 2). On the contrary to this, ‘Gulab Jamun’ mangoes did not exhibit evidence of any influence of MT application on the cellular energy status (i.e., AMP, ADP, ATP, and AEC; Table 2).

**TABLE 2:** Adenosine triphosphate (ATP), adenosine diphosphate (ADP), adenosine monophosphate (AMP), and adenylate energy charge (AEC) in peel and pulp of ‘Langra’ and ‘Gulab Jamun’ mangoes treated with 0 μM (control) or 100 μM (treated) melatonin for 2 h followed by low temperature storage at 5 ± 1°C.

Parameter	Day	Langra				Gulab Jamun			
		Peel		Pulp		Peel		Pulp	
		0 μ M	100 μ M	0 μ M	100 μ M	0 μ M	100 μ M	0 μ M	100 μ M
ATP	0	94.40 ± 3.67 a	94.40 ± 3.67 a	23.10 ± 1.77 a	23.10 ± 1.77 a	99.41 ± 2.04 a	99.41 ± 2.05 a	25.53 ± 2.37 a	25.53 ± 2.37 a
	7	76.50 ± 3.64 b	87.66 ± 3.51 a	19.89 ± 1.20 ab	20.85 ± 1.32 a	87.66 ± 3.51 b	88.67 ± 3.31 b	20.85 ± 1.32 b	21.85 ± 1.54 ab
	14	67.97 ± 4.00 b	75.98 ± 3.18 b	17.00 ± 0.81 bc	19.48 ± 0.78 ab	75.98 ± 3.18 c	74.49 ± 4.05 c	19.48 ± 0.78 b	19.70 ± 0.73 bc
	21	55.50 ± 4.12 c	62.31 ± 3.92 c	15.10 ± 0.89 cd	16.88 ± 0.71 bc	62.31 ± 3.92 d	60.50 ± 3.74 d	16.88 ± 0.71 bc	16.55 ± 0.90 cd
	28	37.14 ± 4.44 d	57.67 ± 4.49 c	12.33 ± 0.91 d	13.85 ± 0.87 c	54.34 ± 3.13 d	53.08 ± 3.013 d	13.85 ± 0.87 c	13.44 ± 0.83 d
ADP	0	56.45 ± 3.19 a	56.45 ± 3.19 a	12.94 ± 1.23 a	12.94 ± 1.23 a	45.38 ± 2.94 a	45.38 ± 2.94 a	11.58 ± 0.86 a	11.58 ± 0.86 a
	7	36.7 ± 1.66 b	44.93 ± 3.50 b	11.15 ± 0.47 ab	12.45 ± 0.90 a	33.24 ± 3.04 b	35.17 ± 3.07 b	8.89 ± 0.66 b	9.86 ± 0.61 ab
	14	29.97 ± 2.03 c	41.60 ± 3.57 b	9.41 ± 0.43 bc	11.52 ± 0.90 a	24.34 ± 2.07 c	28.86 ± 3.82 bc	8.52 ± 0.78 b	9.02 ± 0.79 ab
	21	25.12 ± 1.19 cd	42.33 ± 1.85 b	7.68 ± 0.52 cd	10.67 ± 0.91 a	20.15 ± 2.24 cd	21.45 ± 3.50 cd	6.24 ± 0.53 c	7.40 ± 0.98 bc
	28	18.67 ± 2.11 d	30.51 ± 3.26 c	6.44 ± 0.31 d	10.85 ± 0.47 a	15.71 ± 2.65 d	15.33 ± 1.83 d	5.17 ± 0.58 c	5.50 ± 0.90 c
AMP	0	40.57 ± 2.87 a	40.57 ± 2.87 a	7.74 ± 1.13 a	7.74 ± 1.13 a	38.04 ± 4.64 a	38.04 ± 4.64 a	3.74 ± 0.47 a	3.74 ± 0.47 a
	7	52.11 ± 3.07 ab	58.25 ± 2.44 b	11.48 ± 0.56 b	14.04 ± 0.90 b	58.25 ± 2.44 b	57.11 ± 3.10 b	6.16 ± 0.79 a	6.20 ± 0.98 a
	14	58.65 ± 2.79 bc	67.21 ± 2.69 bc	15.10 ± 0.89 c	16.88 ± 0.71 bc	67.21 ± 2.69 bc	67.98 ± 2.54 bc	16.88 ± 0.71 b	16.55 ± 0.90 b
	21	68.61 ± 4.15 cd	71.93 ± 4.54 cd	17.00 ± 0.81 c	19.48 ± 0.78 cd	71.93 ± 4.54 bc	75.39 ± 5.32 c	19.48 ± 0.78 bc	19.70 ± 0.73 c
	28	79.19 ± 5.95 d	102.09 ± 6.94 d	19.89 ± 1.20 d	20.85 ± 1.32 d	74.89 ± 7.15 c	75.96 ± 6.35 c	20.85 ± 1.32 c	21.85 ± 1.54 c
EC	0	0.64 ± 0.01 a	0.64 ± 0.01 a	0.67 ± 0.02 a	0.67 ± 0.02 a	0.67 ± 0.02 a	0.67 ± 0.02 a	0.77 ± 0.02 a	0.77 ± 0.02 a
	7	0.57 ± 0.02 b	0.58 ± 0.01 b	0.60 ± 0.02 b	0.57 ± 0.01 b	0.58 ± 0.02 b	0.59 ± 0.01 b	0.71 ± 0.01 bc	0.71 ± 0.02 bc
	14	0.53 ± 0.01 c	0.52 ± 0.02 c	0.52 ± 0.01 cd	0.53 ± 0.02 cd	0.53 ± 0.02 bc	0.52 ± 0.02 bc	0.53 ± 0.02 c	0.54 ± 0.01 c
	21	0.46 ± 0.01 d	0.47 ± 0.02 d	0.48 ± 0.02 d	0.47 ± 0.01 d	0.47 ± 0.03 cd	0.45 ± 0.01 cd	0.47 ± 0.02 d	0.46 ± 0.02 d
	28	0.34 ± 0.03 e	0.38 ± 0.01 e	0.40 ± 0.01 e	0.42 ± 0.02 e	0.43 ± 0.01 d	0.42 ± 0.01 d	0.41 ± 0.03 e	0.40 ± 0.01 e

*Measurements were taken on every 7 days of 5 ± 1°C storage followed by 3 days of shelf life at room temperature (25°C, 90–95% RH). Each value is the mean for three replicates. Values followed by different small letters in the same column show a significant difference (p ≤ 0.05) within the same storage period.*

## Discussion

Due to the need for refrigeration to extend shelf life, the occurrence of CI is the major limiting factor that restricts the expansion of marketing for mango fruit. The CI reduction in ‘Langra’ mangoes with 100-μM MT application shown here is in line with previous studies done with peach (27), pomegranate (21), and tomato (22, 23). In accordance with the previous studies, the result of the present study has shown contrasting results for MT application in CI alleviation in cultivars of mango (Figure 1A). Here, ‘Langra’ mangoes have shown maximum CI alleviation with MT application, whereas ‘Gulab Jamun’ mangoes have not exhibited any influence of MT application. The contrasting behavior of ‘Langra’ and ‘Gulab Jamun’ mangoes may be because of differences in the hormonal, physiological, and molecular mechanisms that are responsible for CI occurrence (43).

We recently proposed that ethylene is the important factor that regulates CI in mango cultivars with MT treatment (7). Ethylene biosynthesis arises from higher ACC synthase (*ACS*) and ACC oxidase (*ACO*) gene expressions. During low temperature storage, ACS is suppressed, resulting in lower production of ethylene. However, when fruit was reconditioned at room temperature, a notable rise in ethylene production occurred due to subsequent ACC production, which led to severe signs of CI (43). The results of the present study depict the delayed rise in the climacteric peak of ethylene in concurrence with the respiration peak in ‘Langra’ mangoes in contrast to ‘Gulab Jamun’ mangoes (Supplementary Figure 1). The delayed climacteric peak of ethylene and respiration in MT-treated ‘Langra’ mangoes is supported by the study done with tomato fruit (44). The ‘Gulab Jamun’ mango ethylene pattern with MT application can be related to the banana fruit study done by He et al. (45). This contrasting ethylene behavior within the same species requires further investigation.

Furthermore, in addition to ethylene control of the ripening pattern, there is a synergistic effect on cell wall degrading enzymes that conclude with fruit softening (46). Higher ethylene biosynthesis and signaling promote cell wall degrading enzyme gene expression and enzyme activity that promotes fruit softening during cold storage (47). Insoluble cell wall polymer breakdown into soluble cellulose and pectin results in softening and, thereby, results in that aspect of ripening of fruit (48) through the cell wall modifying enzymes PG, PE, and EGase. With progressive ripening, pectin degradation is accomplished with the hydrolyzing action of PG on the pectic acid α-1,4 glycosidic bond. PG is classified as *exo-*PG and *endo-*PG on the basis of the mode of action. *Exo-*PG degrades pectic chains’ non-reducing free ends and *endo-*PG is responsible for random degradation of the pectic chain.

Internal sites on the glucan backbone of xyloglucan chains are the EGase enzyme action sites that lead to softening of fruit (49). We also observed that the rising activity of PG (*exo-*PG and *endo-*PG) and EGase lead to mango fruit softening in line with the studies done by Zaharah et al. (48) and Chourasia et al. (49)in mangoes. The present study highlights the efficacy of MT application in suppressing PG (Figure 2) activity in addition to higher maintenance of ‘Langra’ mangoes firmness (Figure 1B) and, thereby, delayed senescence with MT application at 5 ± 1°C. Similarly, the suppressed activity of EGase enzyme in peel (Figure 3A) and pulp (Figure 3B) of ‘Langra’ mangoes with the application of MT is consistent with the study done by Zaharah et al. (48), in which nitric oxide was shown to reduce EGase activity and also suppress ethylene production during ripening and, thereby, alleviate CI.

Whereas, demethylation of galacturonyl residues is accomplished through the action of the PE enzyme. In contrast to PG and EGase, the activity of PE (Figures 3E–H) followed a declining pattern over the period of storage. The probable reason for the declining trend of PE in relation to maintenance of firmness in MT-treated ‘Langra’ mangoes is because methyl group removal from polygalacturon will provide more binding sites for PE activity and, thereby, result in high calcium interaction, which would tend to strengthen the rigidity of cell walls (11). The inconsistency in mango cultivars as reported in the present study is the possible cause of CI variability among them. In ‘Langra’ mangoes treated with MT, lower ethylene and ABA biosynthesis accompanied by lower PG, PME, and β-Galactosidase enzyme activities could be responsible for delaying softening (11) and, henceforth, maintaining the firmness of fruit (Figure 1).

During cold storage, the NaCl transcription factor in green bell pepper fruit (50) and *MYB21*/MYB54 transcription factor in pear fruit (44) were responsible for triggering *PLD* gene expression and membrane phospholipids degradation. Moreover, higher ethylene signaling is represented by higher expression of ethylene response factor (*ERF*) genes (51). By triggering ethylene signaling in mango fruit during cold storage, phosphatidylinositol 3-kinase (*PI3K*) gene expression and enzyme activity could be responsible for supplying phosphatidylinositol 3-phosphate (PI3P) for PLD enzyme activity. PLD enzyme activity produces phosphatidic acid (PA), which could trigger cytosolic Ca^2+^ accumulation and promote NADPH oxidase enzyme activity (52–54). By promoting cytosolic Ca^2+^ accumulation, the triggering of calcium−dependent protein kinases (*CDPKs*) could be responsible for promoting shikimate and phenylpropanoid pathway activities for phenol accumulation, which could be responsible for pulp browning in mango fruit during the cold storage *via* polyphenol oxidase (PPO) enzyme activity (54). In addition, PA produced by PLD activity could trigger ethylene signaling by suppressing *CTR1* activity, which serves as a negative regulator of ethylene signal transduction (53).

Along with PLD activity, lipolytic acyl-hydrolase (LAH) enzyme activity would provide free fatty acid substrate for the linolenic acid oxidation pathway *via* either LOX enzyme activity or non-enzymatic peroxidation by ROS, which would give rise to the malondialdehyde (MDA) accumulation (54, 55). The associated membrane deterioration could be responsible for the impedance of H^+^-ATPase and Ca^2+^-ATPase activity, which promotes PLD autocatalytic activity by cytosolic Ca^2+^ accumulation (53, 54). Therefore, maintenance of membrane integrity by employing safe and eco-friendly approaches such as MT application could be beneficial for the maintenance of the energy status of cells and, thereby, would be able to alleviate CI in fruits and vegetables during cold storage. Accordingly, as per the present study, exogenous application of MT to ‘Langra’ mangoes significantly (*p* < 0.05) reduced PLD activity (Figures 4A,B), LOX activity (Figures 4E,F), and MDA content (Supplementary Figure 2) in contrast to ‘Gulab Jamun’ mangoes.

Furthermore, maintenance of membrane fluidity due to higher membrane unSFA/SFA accumulation would be expected to result in the preservation of membrane integrity as represented by lower MDA accumulation. This would be accomplished by means of impeding *PLD* and *LOX* gene expressions and enzyme activities, which have been found to be beneficial for alleviating CI in tomato (23), pomegranate (21), and bell pepper fruits (31) treated with MT. Accordingly, the present work also manifests the effectiveness of 100-μM MT application in CI alleviation in ‘Langra’ mangoes in contrast to ‘Gulab Jamun’ mangoes (Figure 1A). The present discrepancy among the studied cultivars might be because of physiological, hormonal, and molecular mechanisms (43) that somehow regulate chilling tolerance. Conclusively, the MT-based suppression of CI was variable in both of the tested cultivars of mango fruit; whereas ‘Langra’ mangoes were found to better develop chilling tolerance by improving the unSFA/SFA ratio due to suppressed PLD (Figure 4) and LOX (Figure 4) activities, probably because of suppressed *CaPLD* and *CaLOX* gene expressions (31). This suppression of PLD and LOX in MT-treated ‘Langra’ mangoes was further evident by the significantly (*p* < 0.05) lower MDA accumulation (Supplementary Figure 2) (4), which is the end-product of lipid peroxidation.

The loss of antioxidant capacity is also notable with the progression of senescence and ripening, which further adds to the disintegration of membranes. In addition, membrane integrity deterioration during progressive ripening and senescence hampers mitochondrial electron transport system activity and disturbs cellular homeostasis. This situation could be responsible for higher ROS (O_2_^–^. and H_2_O_2_) accumulation, along with lower ATP biosynthesis (9). However, in the present study, the application of MT exhibited a positive response in the alleviation of ROS (Supplementary Figures 3, 4) and thereby was able to maintain antioxidant activity in ‘Langra’ mangoes (Supplementary Figures 5, 6) in contrast to ‘Gulab Jamun’ mangoes.

In addition to triggering membrane deterioration in blueberry fruit treated with nitric oxide, delaying softening could be ascribed to preserving membrane fluidity and integrity resulting from higher ATP-dependent fatty acid-biosynthezing acetyl-CoA carboxylase (ACCase), along with fatty acid unsaturation of *FADs* gene expressions concomitant with lower fatty acid-peroxidizing *LOX* gene expression and enzyme activity (56). In mango fruit treated with 6-benzylaminopurine, suppression of ethylene biosynthesis by lower ACS and ACO enzymes activity, prevention of O_2_^–^ and H_2_O_2_ accumulation, and preservation of membrane integrity, as shown by lower MDA accumulation arising from lower *PLD* and *LOX* genes expression and enzymes activity, could be responsible for retarding fruit softening (57). In addition to preserving membrane integrity, supplying sufficient intracellular ATP could be beneficial for delaying softening *via* support of cell wall biosynthesis and reinforcement (58). During the postharvest period, intracellular ATP is the universal energy currency arising from intracellular ATP biosynthesis. ATP is produced in the glycolysis pathway, the tricarboxylic acid cycle, and the electron transport system, along with the GABA shunt pathway and the energy dissipating alternative oxidase (AOX). Uncoupling proteins (U) activity, energy signaling target of rapamycin (TOR), and sucrose non-fermenting-1 (SNF1)-related protein kinase 1 (*SnRK1*) kinases, accompanied by H^+^-ATPase, Ca^2+^-ATPase, and Mg^2+^-ATPase enzymes activity, are also responsible for alleviating stresses, delaying senescence, and preserving quality in fruits and vegetables during postharvest life (9).

Employing exogenous MT application has been beneficial for alleviating CI in tomato (23) and litchi (30) fruits by promoting intracellular ATP supply by means of promoting H^+^-ATPase, Ca^2+^-ATPase, SDH, and CCO enzyme activities. A similar alleviation with MT application is seen in the present study but with variability between two mango cultivars. This cultivar-dependent CI variability is in accordance with the difference in ATP and ADP accumulation, where ‘Langra’ mangoes treated with MT maintained the energy units in significantly (*p* < 0.05) higher amounts than ‘Gulab Jamun’ mangoes (Table 2). Furthermore, the higher energy status in MT-treated ‘Langra’ mangoes can be briefly explained by the higher activity of energy metabolizing enzymes, namely, H^+^-ATPase (Figures 5E–H) and Ca^2+^-ATPase (Figures 5A–D). The transmembrane potential through H^+^ transportation out from the cell is assisted by H^+^-ATPase, whereas Ca^2+^-ATPase, more properly Ca^2+^ pump, results in the production of ADP and phosphate due to ATP hydrolysis. Additionally, Ca^2+^-ATPase stabilizes the intracellular concentration of Ca^2+^ and thereby maintains the physiological functioning of the plant cells (9). Similarly, in the present work, the higher enzymatic activity of H^+^-ATPase and Ca^2+^-ATPase in MT-treated ‘Langra’ mangoes could be responsible for the better-maintained energy status; hence, the observed maximum chilling tolerance in the cultivar than ‘Gulab Jamun’ mangoes.

Exogenous MT application could be responsible for alleviating CI in mango fruit by preserving membrane integrity *via* suppression of PLD and LOX enzymes activity, protecting the intracellular energy supply *via* promotion of H^+^-ATPase, Ca^2+^-ATPase, SDH, and CCO activities, and preserving cell wall integrity *via* impeding increased of PG, PE, and EGase activities at the epigenetic level by DNA methylation (55, 56), transcriptional level by transcription factors *NACs*/*MYBs* (31, 44, 50), and miRNA (33), and post-translational SUMOylation *via* SUMO E3 ligase SIZ1 (59).

## Conclusion

It can be concluded that chilling tolerance in mango is cultivar-dependent with some variability after pre-storage MT (100 μM) application. MT-treated mangoes of the ‘Langra’ cultivar were found to be CI-tolerant. This chilling tolerance with MT treatment in response to the chilling temperature of 5 ± 1°C in ‘Langra’ mangoes was found to be associated with lower activity of cell wall-modifying enzymes, better-maintained cell membrane, and unsaturated-to-saturated fatty acid ratio, in addition to maintenance of higher cellular energy status in both peel and pulp of the fruit. On the contrary, ‘Gulab Jamun’ mangoes failed to maintain the same preservative metabolisms neither in peel nor in pulp in response to MT application and there was no development of chilling tolerance.

## Data Availability Statement

The raw data supporting the conclusions of this article will be made available by the authors, without undue reservation.

## Author Contributions

RB and SP: conceptualization, methodology, investigation, and writing—original draft preparation. RB: software, validation, and formal analysis. SP: resources, visualization, supervision, and project administration. RB, SP, and MSA: data curation. SP, MSA, MBA, JB, and OF: writing—review and editing. SP and OF: funding acquisition. All authors have read and agreed to the submitted version of the manuscript.

## Conflict of Interest

The authors declare that the research was conducted in the absence of any commercial or financial relationships that could be construed as a potential conflict of interest.

## Publisher’s Note

All claims expressed in this article are solely those of the authors and do not necessarily represent those of their affiliated organizations, or those of the publisher, the editors and the reviewers. Any product that may be evaluated in this article, or claim that may be made by its manufacturer, is not guaranteed or endorsed by the publisher.
