# Supplementary material for: Melatonin Alleviates Chilling Injury Symptom Development in Mango Fruit by Maintaining Intracellular Energy and Cell Wall and Membrane Stability
Source: Front Nutr. 2022 Jun 30;9:936932. doi: 10.3389/fnut.2022.936932 (PMC9280488; doi:10.3389/fnut.2022.936932)
Supplement: Supplementary file 1 [file Table_1.DOCX]

**Supplementary Information**

**Melatonin alleviates chilling injury symptom development in mango fruit by maintaining intracellular energy and cell wall and membrane stability**

**Methodology**

*Ethylene production and respiration rate*

An airtight glass container (800 mL) was used to quantify ethylene production, which was measured in the container’s headspace by piercing the probe of an ethylene analyser (Bioconservacion, Spain) into it. The measurements of the apparatus range from 0 to 100 ppm through the septum fixed to the container lid. Ethylene production was measured after 2 h at 20 ºC, and was expressed as nmol kg^-1^ s^-1^ by using the formula:

$$Ethylene production=\frac{ethylene produced \times headspace volume}{fruit weight \left( \mathrm{kg} \right) \times time (s)}$$

Respiration rate was measured by piercing the probe of an auto gas analyser (PBI Dansensor, Denmark) into the container described for ethylene production, through the rubber septum fixed on the lid of the container. Measurements were taken after 2 h at 20 ºC, and production of CO_2_ was expressed in nmol kg^-1^ s^-1^ by using the following formula:

$$Respiration rate=\frac{\mathrm{CO}_{2} produced \times headspace volume}{fruit weight \left( \mathrm{kg} \right) \times time (s)}$$

*Malondialdehyde (MDA) content*

Malondialdehyde (MDA) was measured according to the procedure described by Jincy et al. [1] Frozen pulp (0.2 g) was homogenized in 0.1 % (w/v) trichloroacetic acid (TCA), followed by centrifugation at 10,000 *g* for 10 min at 4 ºC. The reaction mixture consisted of 0.3 mL supernatant, 1.2 mL of 0.5 % (w/v) thiobarbituric acid (TBA) prepared in 20 % (w/v) TCA, and was incubated for 30 min at 95 ºC. After this time, the reaction was stopped by submerging the tube in an ice bath for 5 min. The cold tubes were centrifuged at 10,000 *g* for 10 min at ambient temperature. The absorbance of the supernatants was measured at 532 nm, from which the nonspecific absorbance at 600 nm was then subtracted. The concentration of MDA was expressed in mM kg^-1^ FW, using 155 mmol^-1^ cm^-1^ as extinction coefficient.

*Superoxide anion (O_2_­­̄^.^) content*

The content of superoxide anion (O_2_^.-^) was determined according to the method of Elstner [2], with some modifications. An extract was prepared using 1 g of fruit tissue (peel or pulp), which was homogenised in 50 mM potassium phosphate buffer (pH 7.8) and centrifuged at 12,000 *g*, at 4 ºC, for 20 min. The supernatant was recovered and used for further analysis.

An assay mixture was prepared, which contained 1 M hydroxylammonium chloride and the supernatant. The mixture was incubated for 1 h at 25 ºC, and 2 mL of ether were then added to prevent chlorophyll interference. After incubation, the solution was centrifuged at 12,000 *g*, for 10 min. The water layer was mixed with 1 mL of 7 mM α-naphthylamine (3:1, v/v solution in glacial acetic acid/water) and 17 mM *p*-aminophenylsulfonic acid (3:1, v/v solution in glacial acetic acid/water). The mixture was incubated for 20 min at 25 ºC, followed by immediate measurement of its absorbance at 530 nm. A sodium nitrate curve was used to calculate O_2_^.-^ production, which was expressed as µM kg^-1^ fresh weight.

*H_2_O_2_ content*

The H_2_O_2_ content was determined according to the method of Sergiev et al. [3], with some modifications. For this, 1 g of fruit tissue (peel or pulp) was homogenised (IKA T18 Digital Ultra-Turrax, Cole-Parmer, India) in 10 mL of a chilled 0.1 % (v/v) trichloroacetic acid (TCA) solution. The homogenate was centrifuged at 12,000 *g*, at 4 ºC, for 15 min, and the supernatant obtained was used to quantify H_2_O_2_. The reaction mixture consisted of 10 mM potassium phosphate buffer (pH 7.0), 1 M KI and the previously obtained supernatant. The reaction was monitored at 390 nm (Specord200plus, Analytik Jena, Germany), and a standard curve of H_2_O_2_ was used to calculate the content of this molecule in the samples. Results were expressed as µM kg^-1^ of fresh weight.

*Trolox equivalent antioxidant capacity (TEAC)*

The TEAC assay was performed with the 2,2’-azino-bis(3-ethylbenzothialozine-6-sulphonic acid) (ABTS) radical (ABTS*^+^), according to the method reported by Re et al. [4], with some modifications. A 7 mM ABTS*^+^ radical solution was initially prepared using an oxidising agent (2.45 mM potassium persulphate). The reaction mixture consisted of the extract and ABTS*^+^ radical solution, which was then incubated for 10 min at 30 ºC. Its absorbance was recorded at 734 nm after the incubation period, and the data used to calculate its scavenging activity, according to the formula described for DPPH. Data is reported as percentage of ABTS radical inhibition.

*Ferric reducing antioxidant power (FRAP)*

The FRAP assay was measured, according to the method of Benzie and Strain [6], with some modifications. FRAP reagent was prepared by mixing 300 mM acetate buffer (pH 3.6), 10 mM 2,4,6-Tris(2-pyridyl)-s-triazine (TPTZ) in 40 mM HCl and 20 mM FeCl_3_ in a 10:1:1 (v/v/v) ratio. The assay mixture contained 2.9 mL of FRAP reagent and 0.1 mL of extract, which were incubated for 30 min at 37 °C. Its absorbance was read at 593 nm after the incubation period, during which an intense blue colour develops as the ferric-tripyridyltriazine complex is reduced to the ferrous state. Absorbances were used to calculate antioxidant potential, and were expressed as g of Trolox equivalents (TE) kg^-1^ fresh weight.

**References**

[1] Jincy M, Djanaguiraman M, Jeyakumar P, Subramanian KS, Jayasankar S, Paliyath G. Inhibition of phospholipase D enzyme activity through hexanal leads to delayed mango (*Mangifera indica* L.) fruit ripening through changes in oxidants and antioxidant enzymes activity. Sci. Hortic. 2017;218:316-25.

[2] Elstner EF. Inhibition of nitrite formation by hydroxyammonium chloride: a simple assay for superoxide dismutase. Anal. Biochem. 1976;70:616–20.

[3] Sergiev I, Alexieva V, Karanov E. Effect of spermine, atrazine and combination between them on some endogenous protective systems and stress markers in plants. C. R. Acad. Bulg. Sci.  1997;51:121-4.

[4] Re R, Pellegrini N, Proteggente A, Pannala A, Yang M, Rice-Evance C. Antioxidant activity applying an improved ABTS radical cation decolorization assay. Free Radic. Biol. Med. 1999;26:1231-7. <https://doi.org/10.1016/s0891-5849(98)00315-3>.

[5] Benzie IF, Strain JJ. The ferric reducing ability of plasma (FRAP) as a measure of “antioxidant power”: the FRAP assay. Anal. Biochem. 1996;239:70-6.

**Figures**

|  |  |
| --- | --- |
|  |  |

**Figure S1.** (a) Rate of ethylene production in ‘Langra’, (b) Rate of respiration in ‘Langra’, (c) Rate of ethylene production in ‘Gulab Jamun’ peel, and (d) Rate of respiration in ‘Gulab Jamun’ with 0 µM (Control) and 100 µM (Treated) melatonin treatment for 2 h followed by low temperature storage 5±1ºC. Readings were taken on every 7 d of 5±1ºC storage followed by 3 d of shelf life at room temperature (25 ºC, 90-95% RH). Each value is the mean for three replicates and vertical bars indicates the standard error. Error bars with striker (*) on the same storage period show significant difference (P < 0.05).

a

**Figure S2.** Malondialdehyde (MDA) content in ‘Langra’ and ‘Gulab Jamun’ mango fruit treated with 0 µM (control) or 100 µM (treated) MT for 2 h followed by storage at 5 ± 1 ºC. Readings were taken on every 7 d of 5 ± 1 ºC storage followed by 3 d of shelf life at room temperature (25 ºC, 90-95% RH). Each value is the mean for three replicates and vertical bars indicates the standard error. Error bars with different small letters on the same storage period show significant difference (P < 0.05).

|  |  |
| --- | --- |
|  |  |

**Figure S3.** O_2_^-^ content in (a) ‘Langra’ peel, (b) ‘Langra’ pulp, (c) ‘Gulab Jamun’ peel, and (d) ‘Gulab Jamun’ pulp treated with 0 µM (control) or 100 µM (treated) melatonin (MT) for 2 h, followed by 28 d of low temperature storage (5 ± 1 ºC) and 3 d of shelf life at room temperature (25 ºC, 90-95% RH). Measurements were taken every 7 d of storage. Each value is the mean of three replicates ± standard error. An asterisk (*) on the same storage period indicates significant differences (P < 0.05).

|  |  |
| --- | --- |
|  |  |

**Figure S4.** H_2_O_2_ content in (a) ‘Langra’ peel, (b) ‘Langra’ pulp, (c) ‘Gulab Jamun’ peel and (d) ‘Gulab Jamun’ pulp treated with 0 µM (control) or 100 µM (treated) melatonin (MT) for 2 h, followed by 28 d of low temperature storage (5 ± 1ºC) and 3 d of shelf life at room temperature (25 ºC, 90-95% RH). Measurements were taken every 7 d of storage. Each value is the mean of three replicates ± standard error. An asterisk (*) on the same storage period indicates significant differences (P < 0.05).

|  |  |
| --- | --- |
|  |  |
|  |  |

**Figure S5.** Trolox equivalent antioxidant capacity (TEAC) in (a) ‘Langra’ peel, (b) ‘Langra’ pulp, (c) ‘Gulab Jamun’ peel and (d) ‘Gulab Jamun’ pulp treated with 0 µM (control) or 100 µM (treated) melatonin (MT) for 2 h, followed by 28 d of low temperature storage (5 ± 1ºC) and 3 d of shelf life at room temperature (25 ºC, 90-95% RH). Measurements were taken every 7 d of storage. Each value is the mean of three replicates ± standard error. An asterisk (*) on the same storage period indicates significant differences (P < 0.05).

|  |  |
| --- | --- |
|  |  |

**Figure S6.** Ferric reducing antioxidant power (FRAP) in (a) ‘Langra’ peel, (b) ‘Langra’ pulp, (c) ‘Gulab Jamun’ peel and (d) ‘Gulab Jamun’ pulp treated with 0 µM (control) or 100 µM (treated) melatonin (MT) for 2 h, followed by 28 d of low temperature storage (5 ± 1ºC) and 3 d of shelf life at room temperature (25 ºC, 90-95% RH). Measurements were taken every 7 d of storage. Each value is the mean of three replicates ± standard error. An asterisk (*) on the same storage period indicates significant differences (P < 0.05).
